# Supplementary material for: Prediction of metabolites associated with somatic mutations in cancers by using genome-scale metabolic models and mutation data
Source: Genome Biol. 2024 Mar 11;25:66. doi: 10.1186/s13059-024-03208-8 (PMC11290261; doi:10.1186/s13059-024-03208-8)
Supplement: Supplementary file 1 — Additional file 1: Figures S1-S9. [file 13059_2024_3208_MOESM1_ESM.docx]

# **Supplementary Figures**

**

 Figure S1. Model statistics of the reconstructed GEMs, including the discarded models.** Number of reactions (pink) and metabolites (purple) across the 1,056 GEMs. These numbers include the GEMs for the entire Eso-AdenoCA samples and six TCGA-LAML samples, which were not considered in this study. These discarded GEMs failed one or more metabolic tasks.


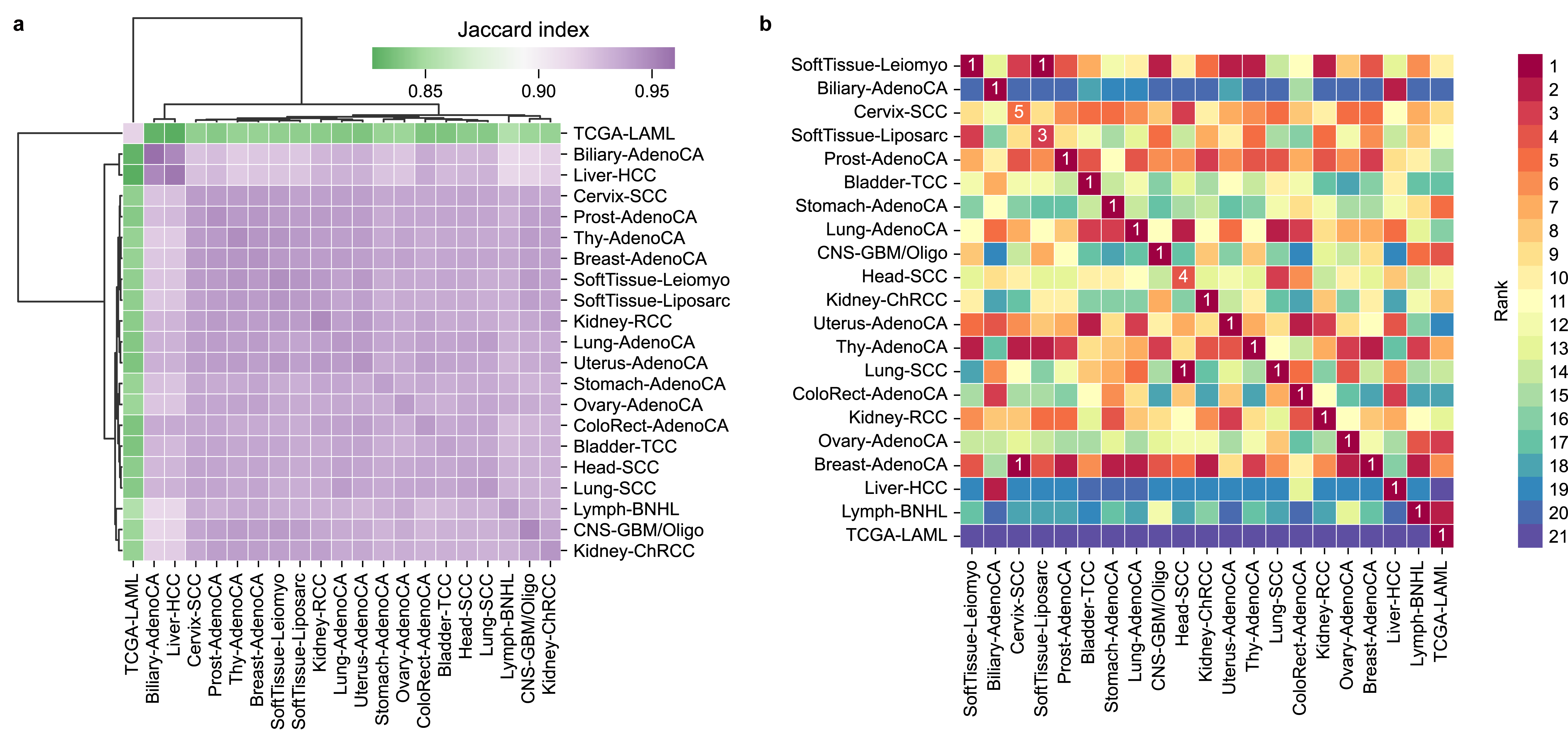


**Figure S2. Pairwise comparison of the 1,033 cancer patient-specific GEMs. a,** Clustered heatmap of Jaccard indices between all pairwise combinations of the patient-specific GEMs (Methods). Color in each cell represents the average of the resulting Jaccard indices. The analysis was conducted using the 1,033 patient-specific GEMs across 21 cancer types. Among the initially reconstructed 1,043 patient-specific GEMs (“Reconstruction of 1,056 cancer patient-specific GEMs across 25 cancer types” in Results), 10 GEMs (i.e., 6 GEMs for Breast-LobularCA, two GEMs for Cervix-AdenoCA, and two GEMs for Skin-Melanoma) were additionally discarded because these three cancer types had fewer than 10 samples (i.e., 10 GEMs), which would result in unreliable average of the Jaccard indices. **b**, Each pairwise comparison of the GEMs is ranked, based on the resulting average Jaccard indices, with ranks ranging from 1 to 21. Rank “1” indicates that the GEMs compared showed the highest similarity.


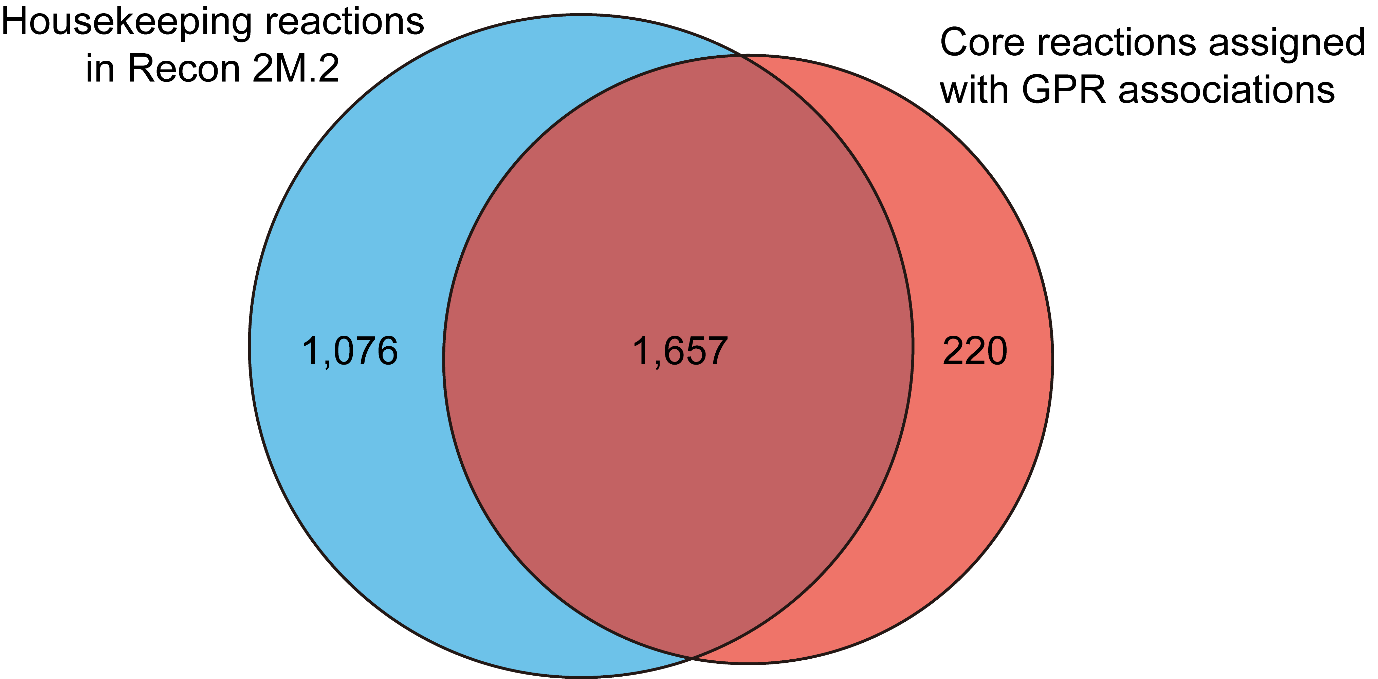


**Figure S3. Number of core and housekeeping reactions in the 1,043 cancer patient-specific GEMs across the 24 cancer types reconstructed in this study.** In the 1,043 cancer patient-specific GEMs reconstructed in this study, 2,804 reactions were considered as ‘core reactions’, which were available in all of the 1,043 GEMs. Among the 2,804 core reactions from the 1,043 GEMs, 1,877 reactions (red circle) were assigned with GPR associations in the GEMs, 1,657 reactions (88.3% of reactions having GPR associations) of which were classified to be housekeeping reactions in normal tissues according to Gatto et al.[1] As a reference, the number of core reactions was 3,510 for the 917 patient-specific GEMs across 13 cancer types developed by Gatto et al. It should be noted that the HMR2[2], which served as a template model for the 917 GEMs developed by Gatto et al., has a greater model size than Recon 2M.2: 8,184 reactions (3,765 genes) versus 5,842 reactions (1,663 genes), respectively.


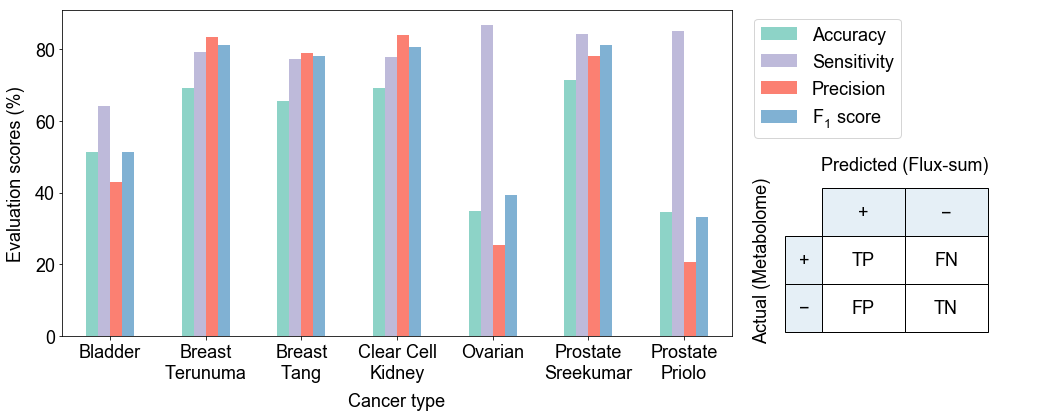


**Figure S4. Evaluation of the capability of flux-sum values to distinguish between normal and cancer samples for five different cancer types in comparison with metabolome data from Reznik et al. [3].** Seven metabolome data retrieved from Reznik et al. [3] present differentially abundant metabolites between normal and cancer samples. The cancer samples represent five different cancer types (i.e., bladder [4], breast [5, 6], kidney [7], ovarian [8] and prostate cancers [9, 10]). These differentially abundant metabolites are the ones showing concentrations significantly different between normal and tumor samples on the basis of two-sided Mann-Whitney U test (Benjamini-Hochberg (BH)-corrected *P* value < 0.05).

Accordingly, personal GEMs for both normal and cancer samples were reconstructed by using RNA-seq data from PCAWG [11] and GTEx [12]. Because RNA-seq data were not available for the samples collected by Reznik et al. [3], we alternatively utilized RNA-seq data for the corresponding samples from PCAWG and GTEx to reconstruct the personal GEMs. As with the metabolome data, metabolites with flux-sum values significantly different between the normal and cancer samples were selected by using the two-sided Mann-Whitney U test. For comparison with the metabolome data, flux-sum values for the same metabolite across multiple compartments were summed into a single value for that metabolite. Also, identifiers of metabolites in the reconstructed GEMs and those from the metabolome data were all converted to MetaNetX IDs (i.e., MNXM IDs) [13]. In the metabolome data, 309 out of 962 metabolites were assigned with MNXM IDs.

In the confusion matrix (right), ‘+’ indicates a metabolite with statistically significant difference (BH-corrected *P* values < 0.05) in the measurement (i.e., metabolome) or prediction (i.e., flux-sum) values between normal and cancer samples; ‘-’ indicates no significant difference in the values between normal and cancer samples. Each metabolite was assigned with true positive (TP), true negative (TN), false positive (FP) or false negative (FN), and accuracy, sensitivity, precision, and F_1_ score were calculated as follows: accuracy, $\frac{TP+TN}{TP+FN+FP+TN}$ $\frac{TP+TN}{TP+FN+FP+TN}$; sensitivity, $\frac{TP}{TP+FN}$; precision, $\frac{TP}{TP+FP}$; and F_1_ score, $\frac{2TP}{2TP+FP+FN}$ .

It should be noted that the flux-sum predictions with low accuracies, for example in “Ovarian” and “Prostate Priolo”, were likely caused by using metabolome data and RNA-seq from different sources as noted above. In case of ‘Prostate Priolo’, the originally reported metabolome data already had inconsistencies (see Figure 4 in Reznik et al.), which may be one reason for poorer prediction performance for ‘Prostate Priolo’; here, “Sreekumar” and “Priolo” are the last name of the first authors for two different studies [9, 10]. For “Ovarian”, tumor heterogeneity as well as the smallest sample number can be additional reasons for the low performance. Nonetheless, the flux-sum values appeared to distinguish between normal and cancer samples for most cancer types.


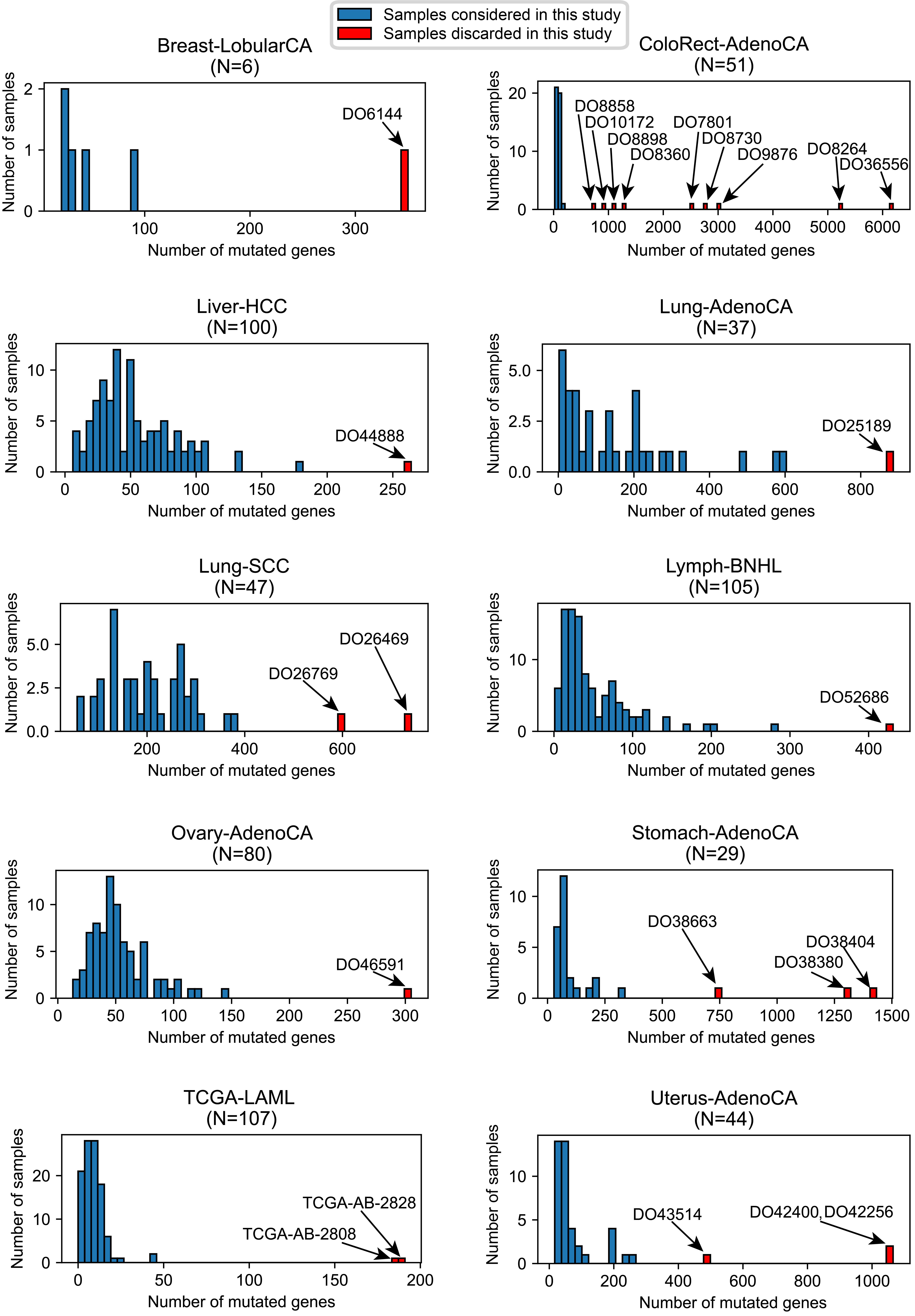


**Figure S5. Selection of cancer samples in this study on the basis of the number of mutated genes.** Cancer samples (red bars) having a total number of mutated genes significantly deviated from other samples in the same cancer type were discarded in this study. Identifiers with ‘DO’ followed by five digits and ‘TCGA-AB’ followed by four digits correspond to donor IDs from PCAWG and TCGA data, respectively.





**Figure S6. Prediction and analysis of metabolite-gene-pathway sets (MGPs) using GEMs that represent the 16 AML samples and the 20 RCC samples. a and b**, (**a**) Number of reactions and (**b**) number of metabolites for the 16 AML GEMs and the 20 RCC GEMs. It should be noted that 17 patient-specific GEMs were initially reconstructed using 17 AML RNA-seq data, but one AML patient-specific GEM was discarded in this study because it did not satisfy all the metabolic tasks (i.e., the incapacity to use L-lysine in mitochondria). **c**, Empirical statistical significance calculated for the number of MG pairs with AUC > 0.7 from the AML samples. Among 420 MG pairs (= 7 mutated genes in the AML samples $\times$ 60 metabolites from Fig. 3b), six MG pairs were randomly selected a million times, and the number of MG pairs with AUC > 0.7 was counted. The red vertical line indicates the number of MG pairs that showed AUC > 0.7 from Fig. 3c,d. **d**, Empirical statistical significance calculated for the number of MG pairs with AUC > 0.7 from the RCC samples. Among 624 MG pairs (= 6 mutated genes in the RCC samples $\times$ 104 metabolites from Fig. 3f), 15 MG pairs were randomly selected a million times, and the number of MG pairs with AUC > 0.7 was counted. The red vertical line indicates the number of MG pairs that showed AUC > 0.7 from Fig. 3g,h. **e and f**, AUC values of metabolites from the metabolome data for (**c**) the 17 AML samples (Fig. 4a-d) and (**d**) the 20 RCC samples (Fig. 4e-h), showing whether the metabolite concentrations significantly change, depending on the mutation of a gene. Left box plots in **c** and **d** indicate metabolites from the metabolome data that are not available in the cancer patient-specific GEMs, and right box plots in **d** indicate the metabolites available in the GEMs. Currency metabolites were not considered in this analysis (Supplementary Table 3). The *P* value was calculated using the two-sided Wilcoxon rank-sum test.


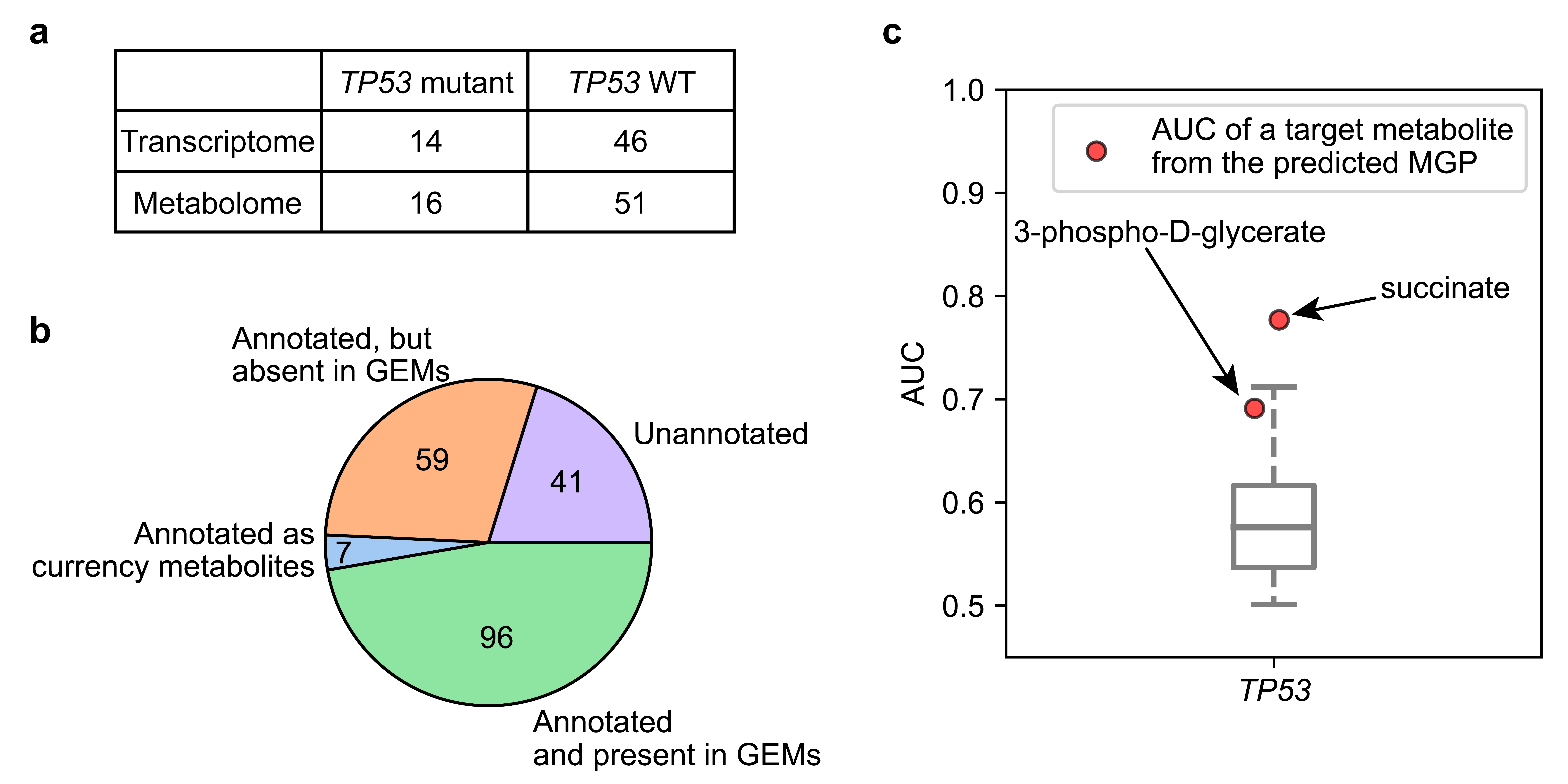


**Figure S7. Analysis of metabolite-gene (MG) pairs from metabolite-gene-pathway sets (MGPs) predicted for the 67 breast cancer samples from Terunuma et al. [5]. a**, Number of transcriptome (gene expression microarray data) and metabolome data from the 67 breast cancer samples. **b**, Classification of the detected peaks from relative quantification of metabolites from the 67 breast cancer samples. **c**, AUC values for two target metabolites involved in the final 21 MGPs (red dots) and 96 metabolites from the metabolome data, each paired with *TP53*. It should be noted that the predicted MGPs also include metabolites (e.g., (R)-mevalonate, (R)-5-phosphomevalonate, and isopentenyl diphosphate) associated with mevalonate pathway, which appeared to be significantly activated as a result of the *TP53* mutation; these predictions are consistent with the previous studies [14, 15].


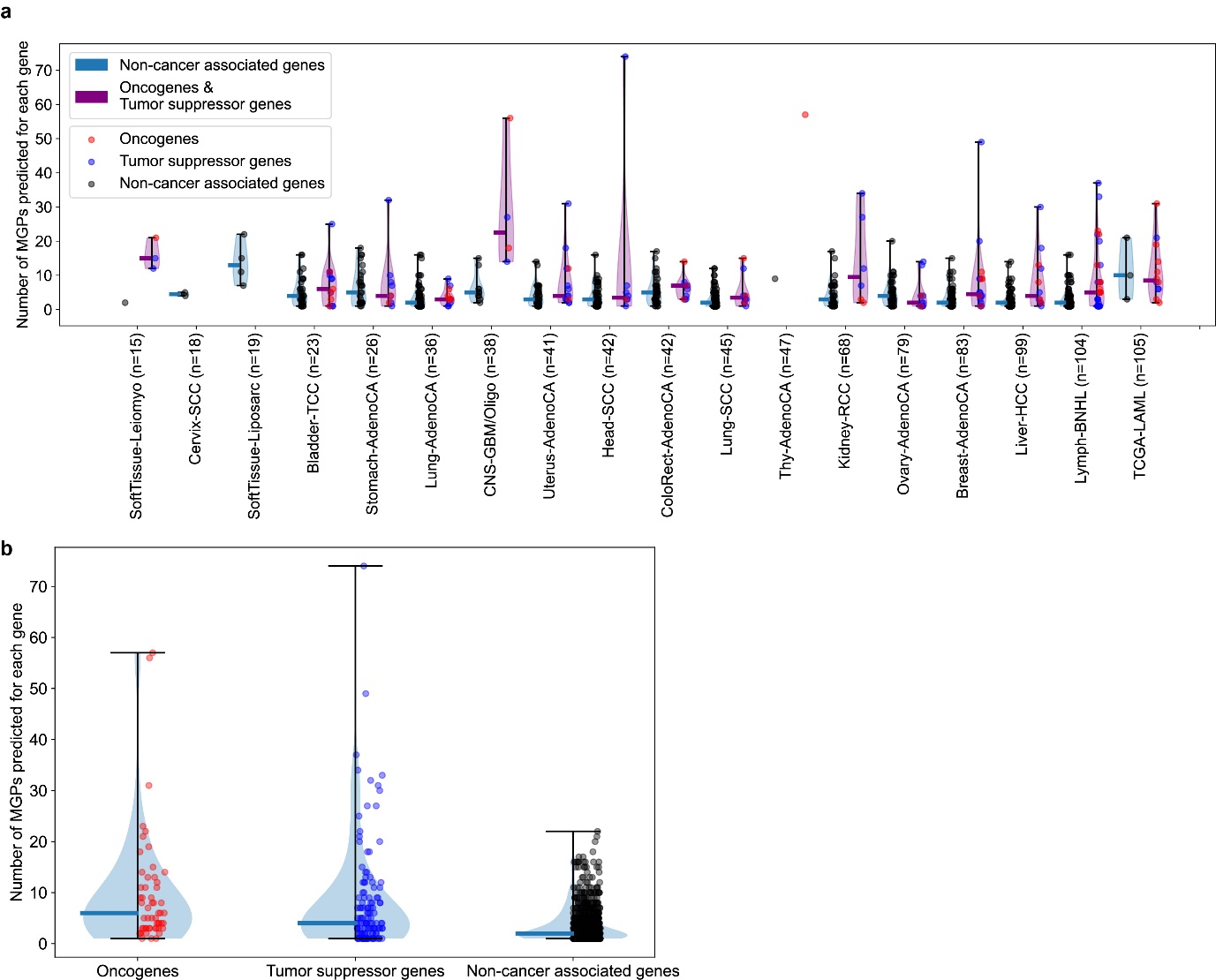


**Figure S8. Number of MGPs associated with each target gene that belongs to oncogene, tumor suppressor gene or non-cancer associated gene. a**, Each data point in the violin plots represents the number of predicted MGPs for each target gene across the 18 cancer types. The body of each violin represents a distribution shape of the number of MGPs. The horizontal lines within each violin plot represent medians. **b**, The three violin plots also represent the same data as (a), but here, the overall number of predicted MGPs is presented for each target gene in terms of a gene type (i.e., oncogene, tumor suppressor gene or non-cancer associated gene).







**Figure S9. Cancer type-specific MGPs associated with four target genes. a-d,** Among the predicted MGPs associated with the top ten target genes across the 18 cancer types (Fig. 5c), MGPs associated with the following four target genes are presented: (**a**) *PBRM1*, the second most frequently mutated gene in Kidney-RCC[16], (**b**) *PIK3CA*, (**c**) *CREBBP*, and (**d**) *FAT1*. The black cell indicates the presence of a MGP that shows the specific combination of a cancer type, a target metabolite, a target pathway and its corresponding submetabolisms. The white cell indicates the absence of such MGP.


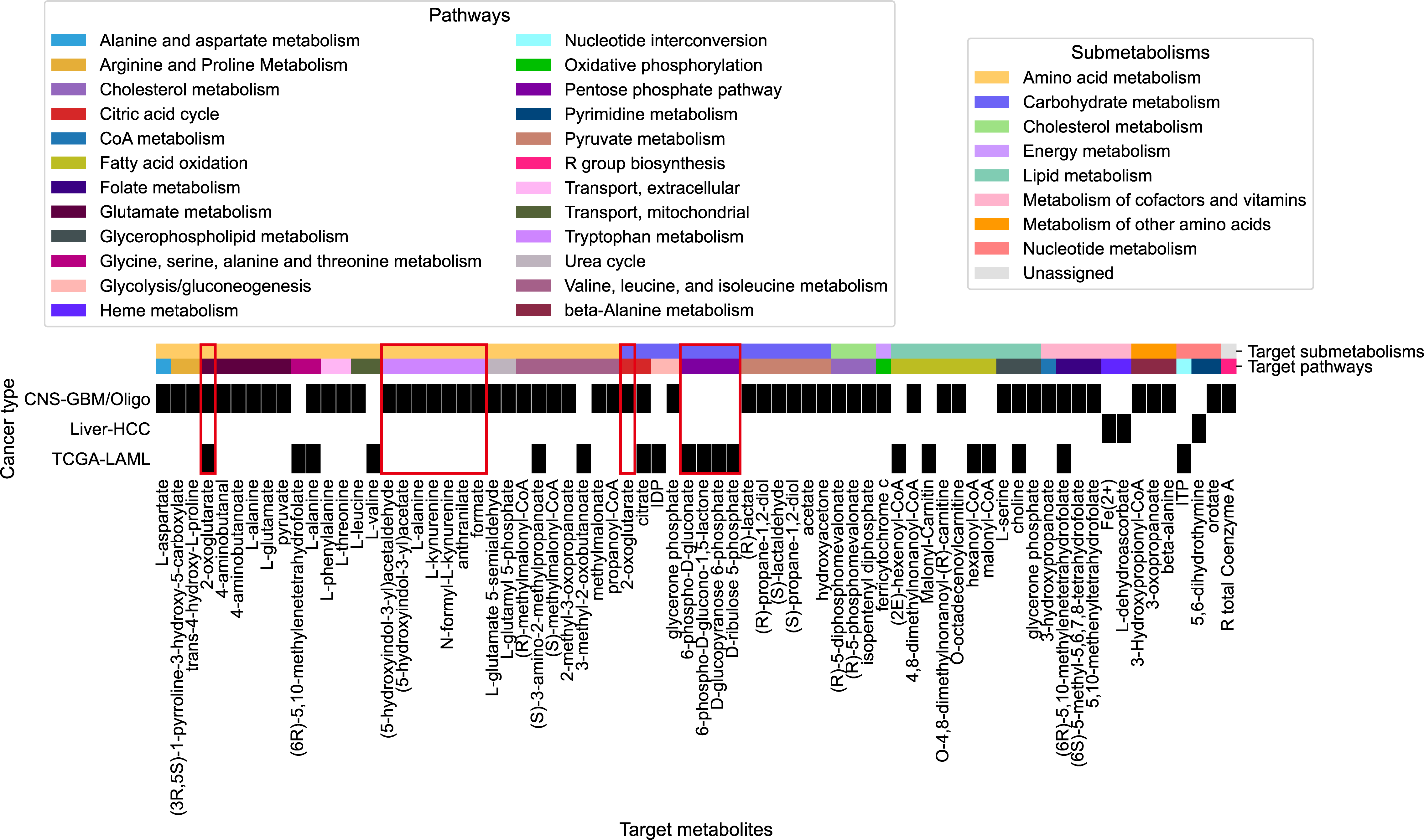


**Figure S10. *IDH*-associated MGPs predicted for CNS-GBM/Oligo, Liver-HCC and TCGA-LAML.** MGPs associated with *IDH (IDH1* and/or *IDH2*), which appeared in CNS-Oligo/GBM, Liver-HCC, and TCGA-LAML, are presented. The black cell indicates the presence of a MGP that shows the specific combination of a cancer type, a target metabolite, a target pathway and its corresponding submetabolisms. The white cell indicates the absence of such MGP. Red boxes indicate the main similarities and differences between CNS-GBM/Oligo and TCGA-LAML with respect to the *IDH*-associated MGPs.


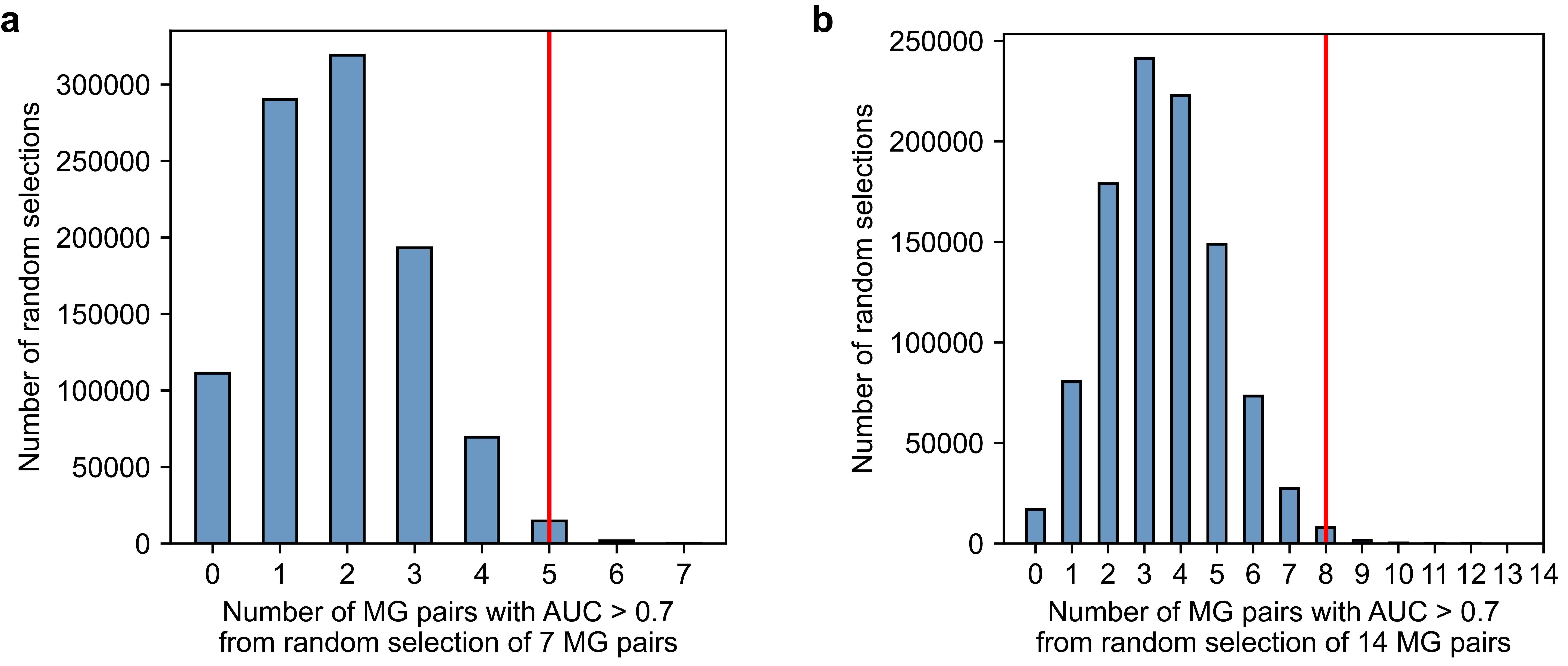


**Figure S11. Evaluating the empirical statistical significance for the number of MG pairs that show AUC > 0.7 AUC when Human1 was used as a template model. a**, Empirical statistical significance calculated for the number of MG pairs with AUC > 0.7 from the AML samples. Among 427 MG pairs (= 7 mutated genes in the AML samples $\times$ 61 metabolites from Fig. 6b), seven MG pairs were randomly selected a million times, and the number of MG pairs with AUC > 0.7 was counted. The red vertical line indicates the number of MG pairs that showed AUC > 0.7 from Fig. 6c,d. (**b**) Empirical statistical significance calculated for the number of MG pairs with AUC > 0.7 from the RCC samples. Among 666 MG pairs (= 6 mutated genes in the RCC samples × 111 metabolites from Fig. 6f), 14 MG pairs were randomly selected a million times, and the number of MG pairs with AUC > 0.7 was counted. The red vertical line indicates the number of MG pairs that showed AUC > 0.7 from Fig. 6g,h.


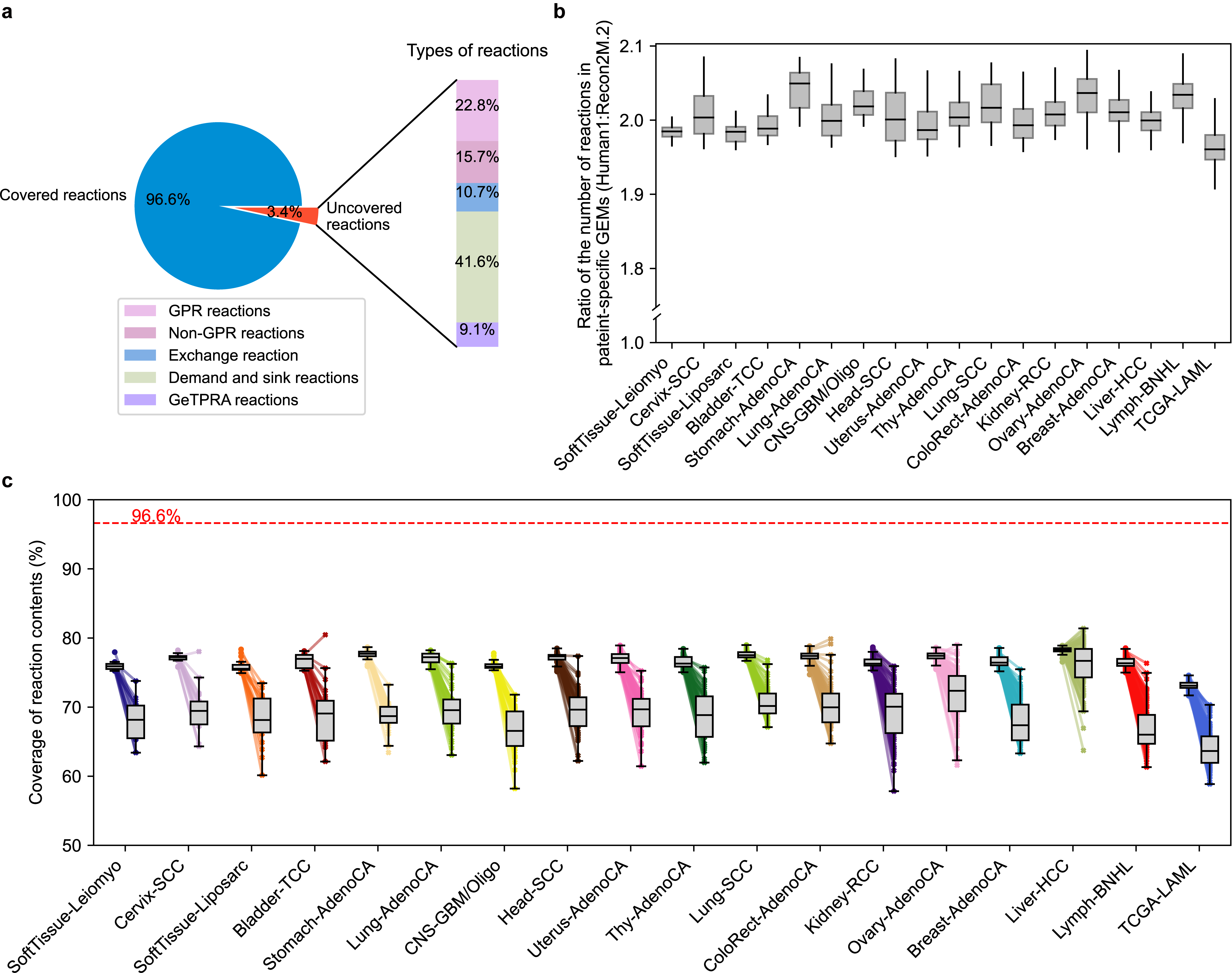


**Figure S12. Comparative analysis of Recon 2M.2 and Human1, and their resulting 956 cancer patient-specific GEMs for the same RNA-seq data. a,** The percentage of reactions in Recon 2M.2 that are covered by Human1. The uncovered reactions contain five distinct reaction types. **b,**  The ratio of the number of reactions in the 956 patient-specific GEMs, reconstructed using Human1 versus Recon 2M.2, for the same RNA-seq data across the 18 cancer types. The patient-specific GEMs derived from Human1 appeared to have almost twice the number of reactions. **c,** The percentage of reactions in the Recon2M.2-based GEMs covered by the Human1-based GEMs (left box plots for each cancer type), and the percentage of “flux-carrying” reactions in the Recon2M.2-based GEMs covered by the Human1-based GEMs (right box plots). For the comparison of flux-carrying reactions, hypermutated samples (Additional file 5: Table S4), which were not considered for the MGP prediction, were also excluded in this analysis. Lines between the two box plots for each cancer type indicate the same samples. The top red line indicates the percentage of reactions in Recon 2M.2 that are covered by Human1 **(a)**.


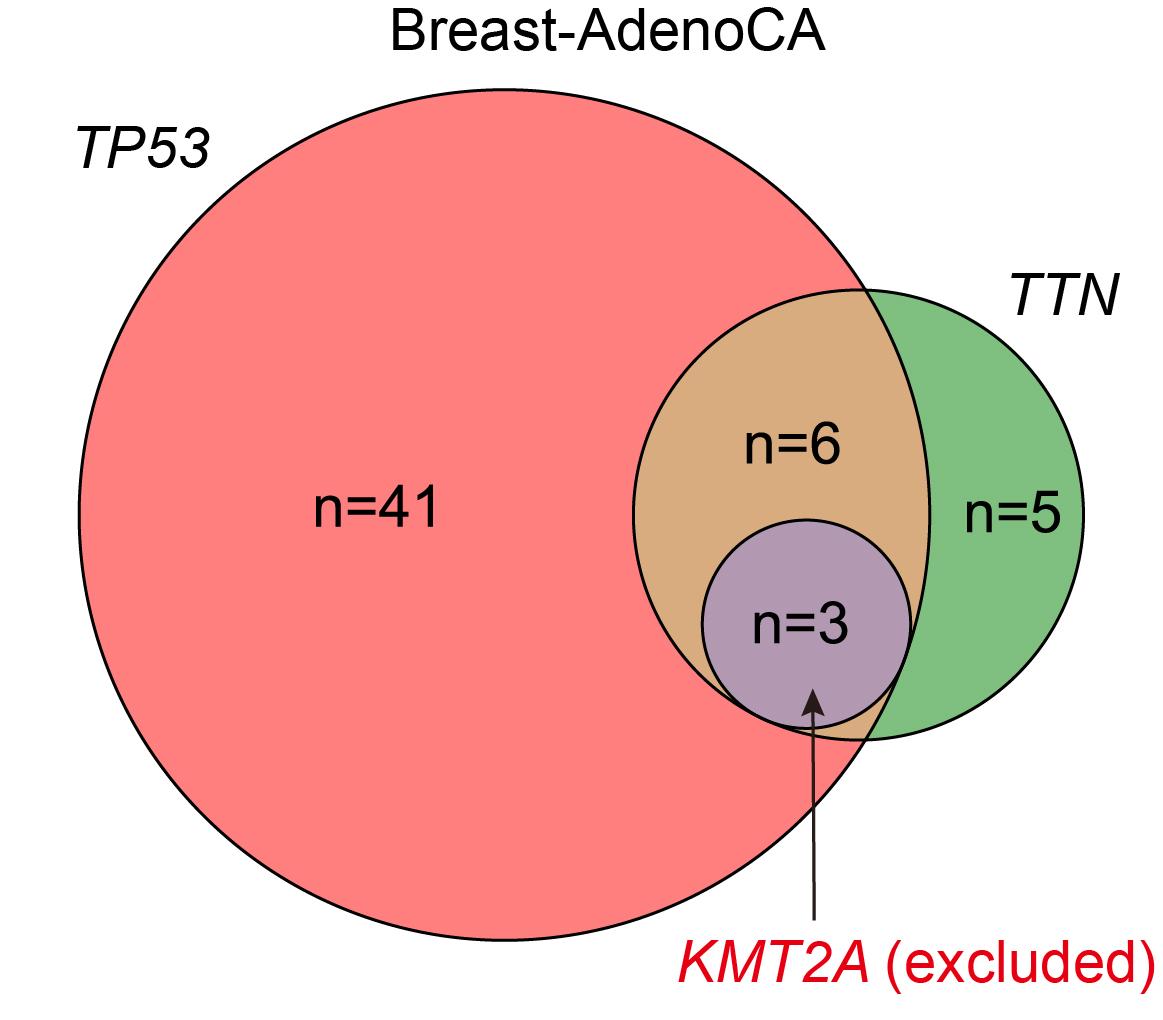


**Figure S13. Illustration of the concept of a ‘subset’ gene mutation with an example of *KMT2A* from Breast-AdenoCA samples.** Breast-AdenoCA from PCAWG mutation data has 50 samples that have *TP53* mutation, and among them, three samples have *KMT2A* mutation; *KMT2A* mutation is a ‘subset’ gene mutation. In such a case, *KMT2A* mutation was not considered in this study because the metabolic effects of the *KMT2A* mutation are not clear as to whether the metabolic effects are caused by *TP53* mutation or *KMT2A* mutation. If at least one sample having *KMT2A* mutation did not have *TP53* mutation, *KMT2A* mutation would have been considered. As a similar example, 14 samples having *TTN* mutation also include *KMT2A* mutation. Likewise, it is not clear whether the metabolic effects come from *TTN* mutation or *KMT2A* mutation, and hence, *KMT2A* mutation was not considered.

# **Supplementary References**

1. Gatto F, Ferreira R, Nielsen J. Pan-cancer analysis of the metabolic reaction network. Metab Eng. 2020;57:51-62.

2. Mardinoglu A, Agren R, Kampf C, Asplund A, Uhlen M, Nielsen J. Genome-scale metabolic modelling of hepatocytes reveals serine deficiency in patients with non-alcoholic fatty liver disease. Nat Commun. 2014;5:3083.

3. Reznik E, Luna A, Aksoy BA, Liu EM, La K, Ostrovnaya I, et al. A landscape of metabolic variation across tumor types. Cell Syst. 2018;6:301-313 e303.

4. Putluri N, Shojaie A, Vasu VT, Vareed SK, Nalluri S, Putluri V, et al. Metabolomic profiling reveals potential markers and bioprocesses altered in bladder cancer progression. Cancer Res. 2011;71:7376-7386.

5. Terunuma A, Putluri N, Mishra P, Mathe EA, Dorsey TH, Yi M, et al. MYC-driven accumulation of 2-hydroxyglutarate is associated with breast cancer prognosis. J Clin Invest. 2014;124:398-412.

6. Tang X, Lin CC, Spasojevic I, Iversen ES, Chi JT, Marks JR. A joint analysis of metabolomics and genetics of breast cancer. Breast Cancer Res. 2014;16:415.

7. Hakimi AA, Reznik E, Lee CH, Creighton CJ, Brannon AR, Luna A, et al. An integrated metabolic atlas of clear cell renal cell carcinoma. Cancer Cell. 2016;29:104-116.

8. Fong MY, McDunn J, Kakar SS. Identification of metabolites in the normal ovary and their transformation in primary and metastatic ovarian cancer. PLoS One. 2011;6:e19963.

9. Sreekumar A, Poisson LM, Rajendiran TM, Khan AP, Cao Q, Yu J, et al. Metabolomic profiles delineate potential role for sarcosine in prostate cancer progression. Nature. 2009;457:910-914.

10. Priolo C, Pyne S, Rose J, Regan ER, Zadra G, Photopoulos C, et al. AKT1 and MYC induce distinctive metabolic fingerprints in human prostate cancer. Cancer Res. 2014;74:7198-7204.

11. The ICGC/TCGA Pan-Cancer Analysis of Whole Genomes Consortium. Pan-cancer analysis of whole genomes. Nature. 2020;578:82-93.

12. GTEx Consortium. The GTEx Consortium atlas of genetic regulatory effects across human tissues. Science. 2020;369:1318-1330.

13. Moretti S, Tran VDT, Mehl F, Ibberson M, Pagni M. MetaNetX/MNXref: unified namespace for metabolites and biochemical reactions in the context of metabolic models. Nucleic Acids Res. 2021;49:D570-D574.

14. Freed-Pastor WA, Mizuno H, Zhao X, Langerod A, Moon SH, Rodriguez-Barrueco R, et al. Mutant p53 disrupts mammary tissue architecture via the mevalonate pathway. Cell. 2012;148:244-258.

15. Parrales A, Thoenen E, Iwakuma T. The interplay between mutant p53 and the mevalonate pathway. Cell Death Differ. 2018;25:460-470.

16. FastQC: a quality control tool for high throughput sequence data. 2010. <http://www.bioinformatics.babraham.ac.uk/projects/fastqc/>. Accessed 30 Jan 2020.
