# Supplementary material for: Prediction of metabolites associated with somatic mutations in cancers by using genome-scale metabolic models and mutation data
Source: Genome Biol. 2024 Mar 11;25:66. doi: 10.1186/s13059-024-03208-8 (PMC11290261; doi:10.1186/s13059-024-03208-8)
Supplement: Supplementary file 12 — Additional file 12. Peer review history. [file 13059_2024_3208_MOESM12_ESM.docx]

**Review history**

**First round of review**

**Reviewer 1**

In this study, the authors investigated the association between mutation and metabolite to identify cancer-associated oncometabolites. For this, mutational data from in-house WES RNA-seq data for AML and RCC samples as well as data from PCAWG, TCGA, ICGC and GTEx were analyzed to associate gene mutation with metabolic pathways that were significantly altered based on GEMs (Recon 2M.2). Many meaningful MGPs were identified and were subsequently validated by an independent GEM template Human-1. This study highlights some important MGPs which could be further investigated in vitro and have potential in clinical translation. The study also proposes a way to integrate genomic data with GEMs which is potentially interesting in the field. Overall, the manuscript is well-written, and all the results and methods are clearly presented.

Major issues:

1. Although the method for transcriptomic integration of the method was developed and probably validated in another study, the fluxes predicted based on GEMs in this study is not validated. Since the author have obtained metabolomic data, they could validate their flux prediction by correlating the flux sum of metabolites with metabolite measurements. I guess this could potentially justify the flux prediction of this study.

Minor issues:

1. Page 5, line 23-26, full names of CNS-GBM and CNS-Oligo should be given when first mention these abbreviations. Or the authors may indicate the cohorts and cancer types in a supplemental table.

2. Page 24, line 45-46. More details can be provided for K-NN imputation for metabolomics data. How many neighbors were selected according to the sample size of the data and why?

3. The resolution of the figures is low. The authors should consider improve it.

**Reviewer 2**

The topic of predicting oncometabolites and metabolic pathways that can help cancer treatment is highly interesting and important. The use of a computational approach leveraging the multi-omics big data is a potentially effective approach to facilitate this goal. The paper is well-written, the logic is clear, and the validation using multi-omics is useful, however, inadequate. Following are a few concerns for the authors to consider to further improve the manuscript before it can be considered for publication.
Major:
1. Different results were generated using different generic human GSMMs. The authors need to thoroughly address this inconsistency to make robust predictions (e.g., by merging the results from the two or more models).
2. Although the authors predicted metabolites and metabolic pathways that can be influenced by specific gene mutation, the authors did not distinguish if the metabolites and metabolic pathways are necessary for survival of the cancer cell, or just passengers that do not have impact for cancer cell survival, which will impede the clinical relevance of the framework proposed. For the very least, this issue should be discussed.
3. The validation is inadequate. For example, in Figs. 3 and 6, some predictions look poor, especially in RCC (Fig. 3h). Empirical statistical significance is needed (e.g., using bootstrapping). Most importantly, The authors should further validate the predicted oncometabolites and metabolic pathways using cell line drug response data, to see if drugs targeting the predicted metabolic pathways indeed have better efficacy in cancer cell killing.
Minor:
4. Page 3 Lines 23-50: also describe the generation and function of L-2-hydroxyglutarate as done with the other known oncometabolites.
5. Page 5 Lines 42-51: need to explain the terms (mass balance, etc). Also, the numbers of metabolic pathways, reactions, and unique metabolites do not make much sense as decimals - change to whole numbers.
6. Page 6 Lines 3-10: "GEMs from the same cancer type tend to be better clustered than those from different cancer types" -- yes for the 5 cancer types highlighted in Fig 1c, but what about the other 19 cancer types? Some clearly don't cluster together well. This should have some kind of quantitative component for how well each cancer type clusters. Furthermore, what is the implication for cancer types that don't cluster well together? Lower quality? Perhaps just greater heterogeneity?
7. Page 6 Lines 40-51: how exactly does flux-sum "quantify the intracellular importance" of a metabolite? Furthermore, have flux-sum approaches been used in mammalian cell studies? Only bacterial studies mentioned.
8. Page 7 Line 12: why is compartment not considered, given that metabolites in different compartments may be participating in different pathways?
9. Page 7 Lines 41-42: define the significance threshold here (e.g., P < 0.05).
10. Page 8 Lines 14-51: what is the rationale of this process? What does "target genes in each target pathway that show corresponding target flux-sum values significantly different from target flux-sum values of other target genes in the same pathway" essentially select for? Are there cases that there are multiple MGP candidates, but nothing is ultimately selected due to non-significance? Why not rank the MGPs by their magnitude of difference in 'target flux-sum' values upon mutation of the target gene, and select them by a uniform cutoff of magnitude of difference?
11. Page 9 Lines 44-47: how were the seven (AML samples) and six (RCC samples) mutated somatic genes determined? Why not use all genes?
12. Page 11 Lines 5-7: need to describe the result in numbers and statistical significance.
13. Page 41: Figure 1; for step 4, add PBRM1's target to the small table showing its target and z-score as well as the Cytoscape figure above it. Only having MGP1/2 doesn't make the selection process visually clear enough.

**Authors’ response to reviewers**

Reviewer reports:

Reviewer #1: In this study, the authors investigated the association between mutation and metabolite to identify cancer-associated oncometabolites. For this, mutational data from in-house WES RNA-seq data for AML and RCC samples as well as data from PCAWG, TCGA, ICGC and GTEx were analyzed to associate gene mutation with metabolic pathways that were significantly altered based on GEMs (Recon 2M.2). Many meaningful MGPs were identified and were subsequently validated by an independent GEM template Human-1. This study highlights some important MGPs which could be further investigated in vitro and have potential in clinical translation. The study also proposes a way to integrate genomic data with GEMs which is potentially interesting in the field. Overall, the manuscript is well-written, and all the results and methods are clearly presented.

[Response] We thank the reviewer for the critical comments and careful review of our manuscript. All of the reviewer’s comments are addressed below.

Major issues:

1. Although the method for transcriptomic integration of the method was developed and probably validated in another study, the fluxes predicted based on GEMs in this study is not validated. Since the author have obtained metabolomic data, they could validate their flux prediction by correlating the flux sum of metabolites with metabolite measurements. I guess this could potentially justify the flux prediction of this study.

[Response] First, as the reviewer noted, flux predictions made using the personal GEMs have been well-established over the past decade. In this study, we used Task-driven Integrative Network Inference for Tissues (tINIT) for the personal GEM generation, and the least absolute deviation (LAD) method for flux prediction. We recently demonstrated that the combination of tINIT and LAD methods generated the flux values tailored to specific cancer types in the best manner among multiple options available [PMID:35782748].

Next, a direct correlation of flux-sum values with metabolite measurements is conceptually invalid because constraint-based flux analysis cannot predict the accumulation of metabolites; this method is based on the pseudo-steady state assumption. Furthermore, the compartmentalized representation of metabolites in the GEMs complicates direct comparisons with metabolome data, which lack compartment information. Also, the numbers of AML and RCC samples generated in this study are not sufficient for a correlation analysis. The objective of this study is to find metabolites associated with mutations across multiple cancer types. Thus, it will be sufficient that the flux-sum values can distinguish metabolic differences between two groups (i.e., wild-type and mutant groups in this study) although the flux-sum values and metabolite concentration values cannot be strictly correlated. Finally, the computational workflow does not just rely on flux-sum values to find MGPs; it also additionally uses information on metabolic pathways and genes to effectively capture metabolic differences between the wild-type and mutant groups. Such rigorous steps ensure the identification of mutation-associated metabolites without knowing a strict correlation between flux-sum values and metabolite concentrations.

Based on this rationale, we performed a new validation for the flux-sum values, which assesses their capability to distinguish between two groups, i.e., normal and cancer groups. For this, we used 7 metabolome datasets (from different studies) from Reznik et al. [PMID: 29396322], covering 5 distinct cancer types. These 7 metabolome datasets contain differentially abundant metabolites between tumor and normal tissues. The results have been newly prepared as Figure S4 in Additional file 1 (page 7 in the revised manuscript). Despite such data heterogeneity, overall, the flux-sum values mostly well-distinguished the cancer samples from their matched normal counterparts, in comparison with the reported metabolome data.

Minor issues:

1. Page 5, line 23-26, full names of CNS-GBM and CNS-Oligo should be given when first mention these abbreviations. Or the authors may indicate the cohorts and cancer types in a supplemental table.

[Response] Full names of the cancer types have now been added in the legend of Fig. 2a. We initially adopted the convention of referencing cancer types using abbreviations, as exemplified in a Nature paper by the Pan-Cancer Analysis of Whole Genomes (PCAWG) Consortium [PMID: 32025007].

2. Page 24, line 45-46. More details can be provided for K-NN imputation for metabolomics data. How many neighbors were selected according to the sample size of the data and why?

[Response] We have added more details regarding K-NN (k-nearest neighbors) imputation for metabolome data in the revised manuscript (page 27). For our study, the K-NN imputation was performed using the “KNN (feature-wise) method” available in MetaboAnalyst. This method imputes missing values based on K-NN analysis of the metabolome data by using concentration profiles of neighbor metabolites. The default value for k in the software is set to 10, and the software does not offer an option to modify this default value. MetaboAnalyst with “KNN (feature-wise) method” has already been widely used [PMID: 34823168, PMID: 34603243, PMID: 37168255; PMID: 35265619]. K-NN with k=10, without using MetaboAnalyst, has also been implemented in noteworthy studies [PMID: 36658343].

3. The resolution of the figures is low. The authors should consider improve it.

[Response] Original figures with high-resolution (i.e., dpi 600) are available by clicking the upper-right of the pages having a figure (e.g., “Click here to access/download; Figure; Figure1.tif”). Figures available in the PDF file from the manuscript system have low resolution.

Reviewer #2: The topic of predicting oncometabolites and metabolic pathways that can help cancer treatment is highly interesting and important. The use of a computational approach leveraging the multi-omics big data is a potentially effective approach to facilitate this goal. The paper is well-written, the logic is clear, and the validation using multi-omics is useful, however, inadequate. Following are a few concerns for the authors to consider to further improve the manuscript before it can be considered for publication.

[Response] We are grateful to the reviewer for the careful review of our manuscript and the critical comments.

Major:

1. Different results were generated using different generic human GSMMs. The authors need to thoroughly address this inconsistency to make robust predictions (e.g., by merging the results from the two or more models).

[Response] For this comment, we have newly prepared “Additional file 11: Dataset S6” and Fig. S12. “Additional file 11: Dataset S6” has a full list of MGPs predicted by using Human1 as a template model. Relevant discussions are newly available on pages 19 and 20 of the revised manuscript.

As expected, the two models generated different sets of the MGPs. Figure S12 was newly prepared to address this inconsistency in greater detail. Figure S12 describes the comparative analysis of 956 patient-specific GEMs across 18 cancer types that were generated by using Recon 2M.2 and Human1 for the same set of RNA-seq data. This new analysis can be summarized as follows:

- Human1 has approximately 2.4 times greater number of unique metabolites and 2.2 times greater number of reactions than Recon 2M.2. Human1 encompasses more than 96% of the Recon2M.2 reactions.

- Different patient-specific GEMs were generated, depending on the use of a generic model for the same RNA-seq data. The Human1-based patient-specific GEMs contain 76.6% reactions of the Recon 2M.2-based GEMs, and the Human1-based GEMs still have about twice as many reactions in total. This indicates that the Human1-based GEMs incorporate a greater number of additional reactions that are absent in the Recon 2M.2-based GEMs for the same patients.

- While the MGPs predicted by using Human1 and Recon 2M.2 are different, using these two different human generic GEMs generated statistically significant results for the predicted MGPs (please see our response for the comment #3 below). More importantly, both models led to the prediction of biologically important MGPs although they differ in what they cover.

2. Although the authors predicted metabolites and metabolic pathways that can be influenced by specific gene mutation, the authors did not distinguish if the metabolites and metabolic pathways are necessary for survival of the cancer cell, or just passengers that do not have impact for cancer cell survival, which will impede the clinical relevance of the framework proposed. For the very least, this issue should be discussed.

[Response] This is a great comment. The issue raised by the reviewer is quite challenging, and calls for further research. We are aware of this concern, which is why we stated in Discussion of the manuscript that “this computational workflow is not intended for an immediate clinical application, for example detecting a cancer biomarker in a person. Rather, it is hoped that the computational workflow and its resulting MGPs serve as the groundwork…” in the original manuscript (pages 21 and 22).

To clarify further, we believe that the predicted MGPs do not necessarily have a direct association with cancer survival, but this also does not mean that the MGPs compromise their clinical relevance.

While it would be intriguing if the MGPs were related to survival, they still offer potential utility as biomarkers even if they are not. Additionally, we are not claiming that the MGPs predicted by this workflow can be directly applied clinically. Rather, we suggest them as groundwork for future studies. Relevant discussion has been further made in the revised manuscript (page 21).

3. The validation is inadequate. For example, in Figs. 3 and 6, some predictions look poor, especially in RCC (Fig. 3h). Empirical statistical significance is needed (e.g., using bootstrapping). Most importantly, The authors should further validate the predicted oncometabolites and metabolic pathways using cell line drug response data, to see if drugs targeting the predicted metabolic pathways indeed have better efficacy in cancer cell killing.

[Response] We would like to thank the reviewer for suggesting empirical statistical significance.

First, as suggested by the reviewer, we have evaluated empirical statistical significance of the MG pairs predicted for the AML and RCC samples. For this, Figures S6c,d and S11 have been newly prepared in Additional file 1 (presented next page). Relevant statements were also made in the revised manuscript (pages 11, 12, and 19).

To calculate the empirical statistical significance, specific numbers of MG pairs were randomly selected a million times (further detailed in the legends of Figures S6c,d and S11). As a result, the chance that any four out of six MG pairs receive AUC > 0.7 is extremely low when using Recon 2M.2 for the AML samples (empirical P value = 0.044; Additional file 1: Fig. S6c). Such statistical significance was also observed for: eight out of the 15 MG pairs with AUC > 0.7 when Recon 2M.2 was used for the RCC samples (empirical P value = 0.018; Additional file 1: Fig. S6d); five out of seven MG pairs with AUC > 0.7 when using Human1 for the AML samples (empirical P value = 0.016; Additional file 1: Fig. S11a); and eight out of 14 MG pairs with AUC > 0.7 when using Human1 for the RCC samples (empirical P value = 0.010; Additional file 1: Fig. S11b).

Further details can be found in the revised manuscript (pages 11, 12, and 19), and Figures S6c,d and S11 in Additional file 1.

Our response to the reviewer’s comments 2 above also applies to the second part of this comment, “validating the predicted oncometabolites and metabolic pathways using cell line drug response data”. The predicted MGPs are not necessarily related to the survival of cancer cells. We believe that this part of the comment is beyond the scope of this study.

Minor:

4. Page 3 Lines 23-50: also describe the generation and function of L-2-hydroxyglutarate as done with the other known oncometabolites.

[Response] Accordingly, we have added the description for the generation of L-2-hydroxyglutarate in the revised manuscript (page 3).

5. Page 5 Lines 42-51: need to explain the terms (mass balance, etc). Also, the numbers of metabolic pathways, reactions, and unique metabolites do not make much sense as decimals - change to whole numbers.

[Response] Accordingly, we have added the description for the terms, and modified the decimals to integers in the revised manuscript (pages 5 and 6) and Additional file 2: Table S1.

6. Page 6 Lines 3-10: "GEMs from the same cancer type tend to be better clustered than those from different cancer types" -- yes for the 5 cancer types highlighted in Fig 1c, but what about the other 19 cancer types? Some clearly don't cluster together well. This should have some kind of quantitative component for how well each cancer type clusters. Furthermore, what is the implication for cancer types that don't cluster well together? Lower quality? Perhaps just greater heterogeneity?

[Response] We believe that the reviewer was were referring to “Fig. 2c”. In the t-SNE plot in Fig. 2c, indeed, while some cancer types were clearly distinguishable, others were less so. In response to the reviewer’s comment, we recognized the need for additional quantitative evaluation of the patient-specific GEMs. To this end, we employed the Jaccard index that quantifies the similarities of two sets; the Jaccard index has been widely used for comparing the reaction contents of two GEMs [PMID: 34194675, PMID: 35339845, PMID: 35754734, PMID: 36719923]. For our analysis, we calculated the Jaccard indices for every possible pair of the patient-specific GEMs, both within the same cancer type and between two different cancer types. Subsequently, the resulting Jaccard indices were averaged for each pair of the GEMs. Except for three cancer types (i.e., Cervix-SCC, SoftTissue-Liposarc, and Head-SCC), greater average Jaccard indices were observed between the GEMs having the same cancer type than those from different cancer types. This analysis additionally suggests a distinct clustering of the GEMs on the basis of their cancer types. We have prepared the results of the Jaccard index analysis as Fig. S2 in Additional file 1 (pages 3 and 4), and this analysis is stated on page 6 of the revised manuscript.

Lastly, for the GEMs that did not cluster well, the inherent metabolic heterogeneity and shared tissues/cells of origins with other cancer types could be reasons.

7. Page 6 Lines 40-51: how exactly does flux-sum "quantify the intracellular importance" of a metabolite? Furthermore, have flux-sum approaches been used in mammalian cell studies? Only bacterial studies mentioned.

[Response] The physical meaning of flux-sum is total production or consumption rate of a given metabolite, essentially representing its turnover rate under a pseudo-steady state condition. Flux-sum of a metabolite is calculated by summing the fluxes of all the reactions that produce or consume a metabolite (as presented in Methods). From a biological standpoint, metabolites that have a higher turnover rate are in high demand, and thus often play pivotal roles in cellular metabolism by serving as key intermediates or end products that are essential for cellular function. Consequently, a higher flux-sum suggests the increased intracellular importance of a metabolite. We have added additional descriptions on flux-sum in the revised manuscript (pages 6 and 7).

For the second part of the comment, flux-sum approaches have also been applied to plants [PMID: 33304454] and mammalian cells [PMID: 28686599, PMID: 32269785]. Additionally, a variant of flux-sum, calculating production rate of a metabolite by introducing artificial sink reactions (for each compartment), has been used in cancer studies [PMID: 33476554, PMID: 33976213]. We have newly cited these studies in the revised manuscript (page 7).

8. Page 7 Line 12: why is compartment not considered, given that metabolites in different compartments may be participating in different pathways?

[Response] Indeed, metabolites within different compartments can participate in different metabolic pathways. However, if multiple metabolic pathways associated with a metabolite appear to be significant, they are all captured as part of MGPs (e.g., target metabolite M-gene G-pathway P1, and target metabolite M-gene G-pathway P2). Also, common metabolome data cannot distinguish metabolites from different compartments. For example, we cannot specify the concentration of the same metabolite in cytosol and nucleus. Consideration of compartment for metabolites would result in multiple MGPs for a single metabolite, each corresponding to different compartments. Validating such MGPs will be challenging because metabolome data have a single value for a single metabolite without compartment information.

9. Page 7 Lines 41-42: define the significance threshold here (e.g., P < 0.05).

[Response] The significance threshold and the methods for calculating P value are described in Methods (“Processing flux-sum values for predicting MGPs”). In response to the reviewer’s comments, we have better clarified this in the revised manuscript (page 8).

10. Page 8 Lines 14-51: what is the rationale of this process? What does "target genes in each target pathway that show corresponding target flux-sum values significantly different from target flux-sum values of other target genes in the same pathway" essentially select for? Are there cases that there are multiple MGP candidates, but nothing is ultimately selected due to non-significance? Why not rank the MGPs by their magnitude of difference in 'target flux-sum' values upon mutation of the target gene, and select them by a uniform cutoff of magnitude of difference?

[Response] This is a great comment. First, this final step of the workflow essentially selects the genes whose effects stand out from the other genes. Our workflow processes mutated genes, as an input, and some of them might have a negligible impact on metabolism. Please note that we did not preselect any mutated gene that is likely to have a significant effect on metabolism in cancer. This would open up the door for finding novel genes associated with oncometabolites, but also, at the same, it may generate false positives (i.e., genes with negligible impact on metabolism). This final process was devised to filter such false positives.

For the reviewer’s comment “Are there cases that there are multiple MGP candidates, but nothing is ultimately selected due to non-significance?”, our response is “yes”.

We have also considered the approach previously that the reviewer suggested. However, the approach had following problems:

- We cannot rank MGPs based on the magnitude of their flux-sum values or similar quantitative values. Each reaction, and thus each metabolite’s flux-sum, has its own intrinsic scale. For example, flux-sum of metabolites involved in central carbon metabolism is relatively greater than those in fatty acid biosynthetic pathway. As a result, with this magnitude-based criterion, some metabolites are always predicted as an MGP, and some others are always not predicted as an MGP.

- Also, rank-based selection (e.g., selecting top 3 among MGP candidates) does not seem to be appropriate because it means that we always have to select, for example, top three MGPs. If the relevant genes are not contributing, they should not be selected, and the rank-based selection does not allow this.

For these reasons, we did not adopt the approach reviewer suggested.

11. Page 9 Lines 44-47: how were the seven (AML samples) and six (RCC samples) mutated somatic genes determined? Why not use all genes?

[Response] Some of our collected samples were subjected to whole exome sequencing (WES), while others were subjected to targeted sequencing. Targeted sequencing covers information on 76 genes, which are considered to be prevalent in AML, and likewise, 148 genes for the RCC samples. As a result, we considered the genes that are covered by both WES and the targeted sequencing. Among the 76 and 148 genes, only seven and six genes met the criteria (e.g., genes having mutations that occur in three or more samples in a cancer type; specified in “Preparation of mutation data from PCAWG WGS data and TCGA WES data” in Methods) for our workflow. We have revised the expression for greater clarity in the revised manuscript (page 10).

12. Page 11 Lines 5-7: need to describe the result in numbers and statistical significance.

[Response] Accordingly, we have added empirical statistical significance in the revised manuscript (page 11).

13. Page 41: Figure 1; for step 4, add PBRM1's target to the small table showing its target and z-score as well as the Cytoscape figure above it. Only having MGP1/2 doesn't make the selection process visually clear enough.

[Response] Accordingly, we have incorporated the reviewer’s suggestion in Figure 1.

**Second round of review**

**Reviewer 2**

The authors have successfully addressed all of the reviewer's concerns, rendering the paper suitable for publication
